# Probiotics for the Primary and Secondary Prevention of *C. difficile* Infections: A Meta-analysis and Systematic Review

**DOI:** 10.3390/antibiotics4020160

**Published:** 2015-04-13

**Authors:** Lynne V. McFarland

**Affiliations:** Department of Medicinal Chemistry, University of Washington, VA Puget Sound Healthcare System, 1660 S. Columbian Way, S-152, Seattle, WA 98108, USA; E-Mail: lvmcfarl@u.washington.edu; Tel.: +1-206-277-1780; Fax: +1-206-764-2935

**Keywords:** probiotics, *clostridium difficile* infections, diarrhea, meta-analysis

## Abstract

*Clostridium difficile* infections are a global clinical concern and are one of the leading causes of nosocomial outbreaks. Preventing these infections has benefited from multidisciplinary infection control strategies and new antibiotics, but the problem persists. Probiotics are effective in preventing antibiotic-associated diarrhea and may also be a beneficial strategy for *C. difficile* infections, but randomized controlled trials are scarce. This meta-analysis pools 21 randomized, controlled trials for primary prevention of *C. difficile* infections (CDI) and four trials for secondary prevention of *C. difficile* recurrences and assesses the efficacy of specific probiotic strains. Four probiotics significantly improved primary CDI prevention: (*Saccharomyces boulardii*, *Lactobacillus casei* DN114001, a mixture of *L. acidophilus* and *Bifidobacterium bifidum*, and a mixture of *L. acidophilus*, *L. casei* and *L. rhamnosus*). None of the tested probiotics significantly improved secondary prevention of CDI. More confirmatory randomized trials are needed to establish if probiotics are useful for preventing *C. difficile* infections.

## 1. Introduction

*Clostridium difficile* infections (CDI) have been a difficult clinical issue for over four decades, with a nearly one-half a million cases per year in the U.S., resulting in 29,000 deaths per year, increased costs of healthcare, outbreaks of CDI in hospitals and long-term care facilities and 83,000 cases of recurrent CDI per year the U.S. [1]. Prevention of CDI has relied on multidisciplinary infection control practices, but guidelines have been found to be difficult to implement globally [2,3].

An innovative strategy to prevent CDI involves using probiotics at the same time antibiotics are given. One recent quasi-experimental study was done in Canada, which gave the mixture of *L. acidophilus*, *L. casei* and *L. rhamnosus* (BioK+) to all patients receiving antibiotics at two hospitals over time and found a significant reduction in the incidence of CDI cases and recurrences at these facilities [4]. Some probiotic strains have been found to be effective for prevention of antibiotic-associated diarrhea (AAD) and for the treatment of CDI [5]. Since CDI accounts for nearly one-third of all AAD cases, this strategy is worth evaluating since CDI persists in impacting our healthcare systems. However, studies of CDI prevention and probiotics have been largely limited to CDI being evaluated as a secondary outcome of AAD studies, leading to underpowerment for CDI outcomes [6]. The technique of meta-analysis allows the pooling of different trials to overcome the low power bias due to the small individual sample sizes. In this paper, randomized, controlled trials of good quality will be pooled to assess probiotic strains for primary and secondary prevention of CDI.

## 2. Results

### 2.1. Initial Screening of Data Search

**Figure 1:**
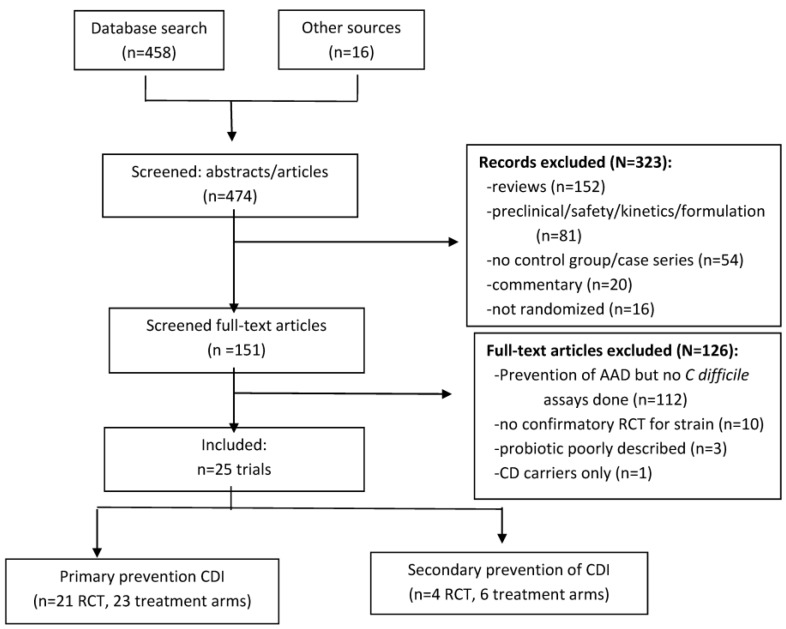
PRISMA flow-diagram of literature search of probiotics for primary or secondary prevention of *Clostridium difficile* infections (CDI).

The literature review yielded 474 abstracts relating to probiotics and CDI that were screened for inclusion. Of those, 323 were excluded after initial screening according to our exclusion criteria (Figure 1): reviews (*n* = 152), pre-clinical animal models or phase two studies for pharmacokinetics, formulation or safety (*n* = 81), no control group or case series (*n* = 54), commentaries (*n* = 20) or not randomized (*n* = 16).

### 2.2. Secondary Screening of Full Articles

Of 151 full-text articles or meeting abstracts screened, 126 were excluded. Most (*n* = 112) were trials for the prevention of AAD that lacked any *C. difficile* outcomes, or the outcome was only for asymptomatic carriage of *C. difficile* (*n* = 1) or the probiotic genus and species were not provided (*n* = 3). Probiotic interventions lacking confirmatory trials (that is, only one trial/probiotic type) were excluded (*n* = 10), as shown in Table 1 [7,8,9,10,11,12,13,14,15,16].

### 2.3. Included Trials

For primary CDI prevention, 21 trials (23 treatment arms) were included [17,18,19,20,21,22,23,24,25,26,27,28,29,30,31,32,33,34,35,36,37] and for secondary CDI prevention, four trials (six treatment arms) were included [38,39,40,41]. Of the 25 randomized controlled trials included, three had multiple treatment arms, [28,37,39] resulting in a total of 29 treatment arms, totaling 4476 participants. Most articles are full-text, peer-reviewed articles (*n* = 23, 92%), but two are available only as meeting abstracts [28,33]. The sample sizes of the trials ranged from 42 to 437, with a mean number per trial of 83 ± 52 in probiotic groups and 82 ± 51 in control groups (*p* = 0.89). Two articles were translated from their original languages into English: one in Hebrew [34] and one in Spanish [23]. The 25 trials were carried out 11 countries: USA (9, 36%), Canada (3, 12%), U.K. (3, 12%), China (2, 8%), Turkey (2, 8%) and one trial (4%) in each: Chile, Finland, Germany, Israel, Italy and Poland. Of the 25 trials included, 19 (76%) tested a single strain of probiotic and six (24%) tested a mixture of probiotic strains. Of the 21 primary prevention trials, the primary outcome of the trials was typically the prevention of AAD (16, 76%), while three (14%) designated the prevention of CDI as a secondary outcome [28,32,34], and two trials (10%) designated CDI as an adverse event associated with their primary outcomes (either prevention of ventilator-associated pneumonia [29] or *Helicobacter pylori* eradication therapy [20]. All four of the secondary CDI prevention trials had the prevention of CDI recurrences as their primary outcome [38,39,40,41].

### 2.4. Study Design

***Degree of blinding in primary prevention***. Of the 21 trials, most (*n* = 18, 86%) were double-blinded (used placebos that were of identical appearance as the probiotic formulation), while three (14%) had open controls, which used either no treatments [20,25] or had an active control with another strain (*L. casei* Shirota) of probiotic as a comparator [31].

***Degree of blinding in secondary prevention***. All four trials had double-blinded, placebo controlled controls.

***Attrition in primary prevention trials****.* As shown in Table 2, attrition ranged from 0%–43% in the 21 trials, drop-outs typically due to adverse events or loss to follow-up. Six trials (29%) reported no attrition, eight (38%) had low attrition frequencies from 1%–10%, four (19%) had moderate attrition from 12%–26%, while three (14%) reported high attrition frequencies (38%–43%). Of the 14 trials with attrition, only two (14%) used Intent-to-Treat (ITT) analysis [20,23], while most (86%) used as-per-protocol (APP) analysis.

***Attrition in secondary prevention trials***. Of the four trials, three reported attrition rates from no attrition [39,41], to 16% attrition [38], but attrition was not reported in one trial [40].

**Table 1:** Prevention of *Clostridium difficile* infections (CDI) for trials with only one study for specific probiotic type.

Probiotic	Eligible Antibiotic Exposures	Daily Dose of Probiotic (cfu/day)	Duration of Probiotic Treatment	Duration Follow-up	CDI in Probiotic Group (%)	CDI in Control Group (%)	Reference
Primary prevention of CDI							
*L. casei* Shirota	nr	6.5 × 10^9^	duration + 1 week	4 weeks	9/76 (0%) ns	1/82 (1.2%)	Wong 2014 [7]
*L. acidophilus*	mixed, 77% beta-lactams	6 × 10^10^	2 weeks	0	0/23 (0%) ns	1/16 (6.2%)	Safdar 2008 [8]
*L. plantarum 299v*	mixed	1 × 10^10^	duration + 1 week	1 week	1/74 (1.3%) ns	0/76 (0%)	Lonnermark 2010 [9]
*Bacillus clausii*	mixed, beta-lactams	4 × 10^9^	duration	6 weeks	0/162 (0%) ns	1/160 (0.6%)	Destura 2008 [10]
*C. butyricum* 588	mixed, 87% beta-lactams	1–4 × 10^7^	6 days	0	0/83 (0%) ns	0/27 (0%)	Seki 2003 [11]
*L rhamnosus* (strains E/N, Oxy, Pen)	mixed, mostly pen and ceph	4 × 10^10^	duration (x = 8 day)	2 weeks	3/120 (2.5%) ns	7/120 (5.8%)	Ruszczynski 2008 [12]
*L. rhamnosus* GG *+L. acido. La5 + Bifido. lactis* Bb12	mixed, nr types	5 × 10^10^	2 weeks	0	0/34 (0%) ns	1/29 (3.4%)	Wenus 2008 [13]
*L. acidophilus (CUL 60 and CUL 21) + Bifido. bifidum CUL20 +Bifido. lactis CUL34*	mixed, 21% single, 70% pen	6 × 10^10^	3 weeks	10 weeks	12/1470 (0.8%) ns	17/1471 (1.2%)	Allen 2013 [14]
VSL#3	mixed, 75% pen	9 × 10^11^	duration + 1 week	3 weeks	0/117 (0%) ns	0/112 (0%)	Selinger 2013 [15]
Secondary prevention of CDI							
*L. plantarum* 299v	mixed	5 × 10^10^	5.4 weeks	4.5 weeks	4/11 (36%) recurred	6/9 (67%)	Wullt 2003 [16]

Abbreviations: Bifido., *Bifidobacterium*; C., *Clostridium*; cfu, colony-forming unit; L., *Lactobacillus*; ns, not significant; VSL#3, contains *Bifido. breve*, *Bifido. longum*, *Bifido. infantis*, *L. acidophilus*, *L. plantarum*, *L. casei*, *L. bulgaricus*, *Strept. thermophilus*; x, mean.

**Table 2:** Study design description for primary prevention of *C. difficile* infections from studies of Probiotics for the Prevention of Antibiotic-associated diarrhea.

Enrolled population	% Attrition	Single or Multiple Types of Inciting Antibiotics	Most Common Type of Antibiotic	Type(s) of Infections	Reference
adults, I	43	59% multiple	36% cepha	mixed, nr	Surawicz 1989 [17]
adults, I	38	82% multiple	beta-lactams	mixed, nr	McFarland 1995 [18]
elderly, I	4.2	nr	nr	nr	Lewis 1998 [19]
adult, O	3.3	100% multiple	amox and clarithromycin	*H. pylori* infections	Duman 2005 [20]
pediatric, I&O	8.5	nr	41% cepha	68% resp, 29% otitis media	Kotowska 2005 [21]
adults, I	0	nr	83% beta-lactams	nr	Can 2006 [22]
adults, O	4.6	100% single	100% amox	88% resp	Bravo 2008 [23]
adults, I	26	69% single	mixed, nr	nr	Pozzoni 2012 [24]
pediatric, I	15	nr	52% cepha	resp	Shan 2014 [25]
pediatric, O	28.7	nr	66% amox	74% otitis media, 26% resp	Arvola 1999 [26]
adults, I	11.6	nr	69% beta-lactams	nr	Thomas 2001 [27]
adults, I	0	nr	cepha	nr	Miller 2008a [28]
adults, I	0	69% single	50% cepha	nr	Miller 2008b [28]
adults, I	5.5	only 34% with VAP on abx	nr	pneumonia	Morrow 2010 [29]
adults, I	19	61% single	66% amox or cepha	49% resp	Hickson 2007 [30]
adults, I	0	nr	60% amp or cepha	80% resp or GU	Dietrich 2014 [31]
elderly, I	8	nr	nr	nr	Plummer 2004 [32]
adults, I	0	nr	mixed	nr	Rafiq 2007 [33]
adults, I	0	nr	48% ceph	nr	Stein 2007 [34]
adults, I	0	nr	59% quinolones	92% resp	Beausoleil 2007 [35]
adults, I&O	7.4	nr	78% beta-lactams	39% resp	Sampalis 2010 [36]
adults, I	9	nr	41% cepha	47% resp	Gao 2010a [37]
adults, I	7	nr	37% cepha	47% resp	Gao 2010b [37]

Abbreviations: amox, amoxicillin; amp, ampicillin; cepha, cephalosporin; GU, genital-urinary infections; I, inpatient; nr, not reported; O, outpatient; resp, respiratory infections; VAP, ventilator-associated pneumonia.

### 2.5. Patient Population

***Primary prevention trials.*** Most of the 21 trials (15, 71%) were done at one hospital, while six (29%) were done at multiple sites (hospitals and/or clinics) [18,20,21,30,32,36]. Most (*n* = 16, 76%) enrolled inpatients, three (14%) of the trials enrolled only outpatients and two (9%) had a mixture of inpatients and outpatients. As shown in Table 2, most of the 21 trials enrolled adult participants (n=18, 86%) and three (14%) enrolled children [21,25,26], and all trials included both genders. Race or ethnicity was not reported in most clinical trials.

***Secondary prevention trials.*** Most of the four trials were done at multiple sites: three sites [38,41] or four sites [39], while one trial was done at one site [40]. Three trials enrolled both inpatients and outpatients [38,39,40], but one trial did not report the type of patient enrolled [41]. All four trials enrolled only adult patients. Two trials enrolled only patients with recurrent CDI [39,41], while two enrolled patients with either incident or recurrent CDI [38,40].

### 2.6. Antibiotic Exposure

***Primary prevention trials***. As shown in Table 2, the types of antibiotic exposures varied widely from single antibiotics to multiple types. Of the 21 trials, only seven reported if single or multiple antibiotics were prescribed, most (88%) had a mix of single and multiple antibiotics. One trial enrolled patients with only amoxicillin use [23]. The most common types of antibiotic exposure were beta-lactams including penicillins and cephalosporins. Of the 21 trials, 11 (52%) reported the type of infection requiring antibiotics, which was most commonly for respiratory infections.

***Secondary prevention trials***. Of the four trials, only one reported the types of inciting antibiotics, but none reported the original disease indication for the antibiotics. In this one trial, 31% were single antibiotics and 69% were multiple types, with the most common type being cephalosporins [38].

### 2.7. Interventions

***Probiotics in primary CDI prevention trials***. Details of the intervention for the 21 RCT (23 treatment arms) for the primary prevention of CDI are given in Table 3. Five different types of probiotics were described in the 21 trials: three single-strain probiotics (*Saccharomyces boulardii* CNCM I-745 (*S. boulardii*), *Lactobacillus rhamnosus* GG, *L. casei* DN114001)) and two types of probiotic mixtures: (*L. acidophilus* and *Bifidobacterium bifidum)* and (*L. acidophilus* CL1285 and *L. casei* LBC80R and *L. rhamnosus* CLR2 (La+Lc+Lr)). Newer strain designations for several probiotics and the retrospective review of older studies may have used different strain designations, but were, in fact, the same strain. The most recent strain designations are used in this study. The most current strain designation for *S. boulardii* is CNCM I-745, the registration number at the Pasteur Institute [42], but older studies also refer to this strain as *S. boulardii* lyo, or *S. boulardii*, with no strain designation or by the brand name “Florastor”. *L. casei* DN114001 is also cited as the brand name “Actimel”. The mixture of *L. acidophilus* CL1285 and *L. casei* LBC80R and *L. rhamnosus* CLR2 is also cited as the brand name “Bio K+” [43].

**Table 3:** Characteristics of probiotic and control treatments and rate of *C. difficile* infections (CDI) by group.

Probiotic	Daily Dose (cfu/d)	Formulation	Duration Treatment	Follow-up (weeks)	CDI in Probiotic	CDI in Controls	Power	Reference
*S. boulardii*	2 × 10^10^	capsules	duration + 2 weeks	0	3 (2.6%)	5 (7.8%)	26.5%	Surawicz 1989 [17]
*S. boulardii*	3 × 10^10^	capsules	duration + 3 days	7	3 (3.1%)	4 (4.2%)	2.6%	McFarland 1995 [18]
*S. boulardii*	4.5 × 10^9^	capsules	duration (x = 7 days)	0	5 (15%)	3 (8.3%)	7.2%	Lewis 1998 [19]
*S. boulardii*	1 × 10^10^	capsules	duration (x = 2 weeks)	4 days	0 (0%)	1 (0.5%)	3.3%	Duman 2005 [20]
*S. boulardii*	1 × 10^10^	wafers	duration (x = 1 week)	0	3 (2.5%)	10 (7.9%)	35.6%	Kotowska 2005 [21]
*S. boulardii*	1 × 10^10^	capsules	duration	4	0 (0%)	2 (2.6%)	9.1%	Can 2006 [22]
*S. boulardii*	1 × 10^10^	capsules	12 days	9 days	0 (0%)	0 (0%)	--	Bravo 2008 [23]
*S. boulardii*	1 × 10^10^	capsules	duration + 7 days	12	3 (2.8%)	2 (2%)	3%	Pozzoni 2012 [24]
*S. boulardii*	1 × 10^10^	powder	duration (x = 2 weeks)	2	1 (0.7%)	8 (5.6%)	51.9%	Shan 2014 [25]
*L. rhamnosus* GG	4 × 10^10^	capsules	duration (x = 7–10 day)	12	1 (1.6%)	1 (1.7%)	10%	Arvola 1999 [26]
*L. rhamnosus* GG	2 × 10^10^	capsules	2 weeks	1	2 (1.5%)	3 (2.2%)	2.7%	Thomas 2001 [27]
*L. rhamnosus* GG	4 × 10^10^	capsules	duration (x = 2 weeks)	4	4 (4.2%)	7 (7.4%)	9.2%	Miller 2008a [28]
*L. rhamnosus* GG	1.2 × 10^11^	capsules	duration (x = 2 weeks)	4	2 (1.3%)	0	11.2%	Miller 2008b [28]
*L. rhamnosus* GG	4 × 10^9^	capsules	duration (x = 15 day)	0	4 (5.8%)	13 (18.6%)	52.9%	Morrow 2010 [29]
*L. casei DN 114001*	2 × 10^10^	drink	duration + 1 week	4	0 (0%)	9 (17%)	81%	Hickson 2007 [30]
*L. casei DN 114001*	2 × 10^10^	drink	duration (x = 6 days)	0	0 (0%)	3 (10%)	21.3%	Dietrich 2014 [31]
*L acidophilus +Bifido. bifidum*	2 × 10^10^	capsules	20 d	0	2 (2.9%)	5 (7.2%)	11.5%	Plummer 2004 [32]
*L acidophilus +Bifido. bifidum*	cfu nr (3g/day)	capsules	duration or LOS	0	5 (11%)	22 (40%)	88.0%	Rafiq 2007 [33]
*L acidophilus +Bifido. bifidum*	6 × 10^9^	capsules	3 weeks	0	3 (14.3%)	1 (4.8%)	7.2%	Stein 2007 [34]
*L. acidophilus* CL1285 *+ L. casei* LBC80R *+ L. rhamnosus* CLR2	5 × 10^10^	milk	duration (x = 7–8 day)	3	1 (2.3%)	7 (15.6%)	44.2%	Beausoleil 2007 [35]
*L. acidophilus* CL1285 *+ L. casei* LBC80R *+ L. rhamnosus* CLR2	5 × 10^10^	milk	duration + 5 days	3	1 (0.5%)	4 (1.8%)	12.5%	Sampalis 2010 [36]
*L. acidophilus* CL1285 *+ L. casei* LBC80R *+ L. rhamnosus* CLR2	5 × 10^10^	capsules	duration + 5 days	3	8 (9.4%)	20 (23.8%)	64%	Gao 2010a [37]
*L. acidophilus* CL1285 *+ L. casei* LBC80R *+ L. rhamnosus* CLR2	1 × 10^11^	capsules	duration + 5 days	3	1 (1.2%)	20 (23.8%)	99.2%	Gao 2010b [37]

Abbreviation: Bifido., *Bifidobacterium*; CDI, *C. difficile* infections; cfu, colony-forming units; L., *Lactobacillus;* LOS, length of stay; nr, not reported; S., *Saccharomyces*; x, mean.

The daily dose of probiotics varied widely from a lower daily dose in three treatment arms (4–6 × 10^9^) [19,29,34] to higher doses ranging from 1–12 × 10^10^ colony-forming units (cfu) per day, while one study did not report their daily dose by cfu/d [33].

Most of the 23 treatment arms used a capsule formulation (74%), while four (17%) were given in milk or other drinks, or as powder (4%) or in wafers (4%).

Probiotics were given in conjunction with the antibiotics (usually started within 48–72 h of the antibiotic) and continued for either the duration of the antibiotic (12 treatment arms, 52%) or continued for 3–14 days after antibiotics were discontinued (7 arms, 30%). Four treatment arms gave the probiotic for a prescribed period (ranging from 14–21 days), regardless of the duration of antibiotics [23,27,32,34].

The duration of follow-up post-antibiotic and probiotic intervention ranged from 0–90 days. Eight (35%) of the treatment arms did not follow patients after the intervention had been discontinued. Most trial arms followed patients for 2–4 weeks (9 arms, 39%), or 1 week (2 arms, 9%) or for only four days (1, arm, 4%), while three (13%) had prolonged follow-up periods from seven to 12 weeks [18,24,26].

As CDI was usually a secondary outcome, not all enrolled trial participants were assayed for *C. difficile*, most trials tested for *C. difficile* when participants developed diarrheal symptoms, but not all trials successfully assayed all participants with diarrhea, nor provided data on the number of participants tested for *C. difficile*. One trial planned *a priori* to assay for *C. difficile* at enrollment, at the end of the intervention and end of follow-up, and successfully assessed 133 (69%) of trial participants, regardless of diarrheal symptoms [18]. Only three other trials reported the frequency of testing for *C. difficile* (done only if diarrhea developed), which was in a limited number of participants: *n* = 16 [20] or *n* = 46 [36], but one study only tested 50% (4/8) participants with diarrhea [23].

***Probiotics in secondary CDI prevention trials***. As shown in Table 4, four of six treatment arms tested a single strain of yeast (*S. boulardii*) [38,39] and two treatment arms tested a single strain of bacteria (*L. rhamnosus GG*) [40,41]. The three treatment arms in one trial combined *S. boulardii* or placebo in three separate antibiotic adjunctive treatments [either low dose vancomycin (500 mg/day), high dose vancomycin (2 g/day) or metronidazole (1 g/day)] [39]. The doses of vancomycin or metronidazole adjuncts were not controlled in the other three trials and were under the discretion of the patient’s primary provider. The daily dose of the probiotic varied from 2–3 × 10^10^/day [38,39] to 3 × 10^11^ [41], but daily dose was not provided in one trial [40]. Five of the treatment arms had a capsule formulation, while one used a probiotic yogurt [40]. The duration of probiotic intervention varied from 3–4 weeks, except in one trial that gave the intervention during adjunctive antibiotic therapy (typically 10–14 days), then extended the intervention for another three weeks [41]. The duration of follow-up was usually four weeks post-intervention, except for one trial that followed patients for 8.6 weeks [41].

**Table 4:** Secondary prevention by probiotic type for treatment of *Clostridium difficile* infections (CDI).

History of CDI	Pop-ulation	Type of controls	Adjunctive therapy (daily dose)	Probiotic	Probiotic daily dose (cfu/day)	Duration treated (follow-up)	Frequency CDI recurrences in probiotic	Frequency CDI recurrences in controls	Power (%)	Reference
I/R	124 adults, In & Out	placebo	V or M (varied)	*S. boulardii*	3 × 10^10^	4 weeks (4 weeks)	15/57 (26.3%)*	30/67 (44.8%)	49.5	McFarland 1994 [38]
R	83 adults, In & Out	placebo	V (500 mg)	*S. boulardii*	2 × 10^10^	4 weeks (4 weeks)	23/45 (51%)	17/38 (44.7%)	5.3	Surawicz 2000a [39]
R	32 adults, In & Out	placebo	V (2 g)	*S. boulardii*	2 × 10^10^	4 weeks (4 weeks)	3/18 (17%)*	7/14 (50%)	35.9	Surawicz 2000b [39]
R	53 adults, In & Out	placebo	M (1g)	*S. boulardii*	2 × 10^10^	4 weeks (4 weeks)	13/27 (48%)	13/26 (50%)	3.3	Surawicz 2000c [39]
I/R	25 adults, In & Out	placebo	V (nr) M (nr)	*L rhamnosus* GG	nr	3 weeks (4 weeks)	4/11 (36.4%)	5/14 (35.7%)	5.7	Pochapin 2000 [40]
R	15 adults	placebo	20% V (nr) 80% M (nr)	*L rhamnosus* GG + inulin	3 × 10^11^	duration abx + 21 days (8.6)	3/8 (37.5%)	1/7 (14.3%)	5.3	Lawrence 2005 [41]

* *p* < 0.05, Abbreviations: abx, antibiotics; CDI, *Clostridium difficile* infection; I, initial CDI episode; In, inpatient; L., *Lactobacillus*; M, metronidazole; Md, median; nr, not reported in paper/abstract; Out, outpatient; R, recurrent CDI; S., *Saccharomyces*; V, vancomycin.

### 2.8. Pooled Efficacy of Probiotics for Primary CDI Prevention

**Figure 2:**
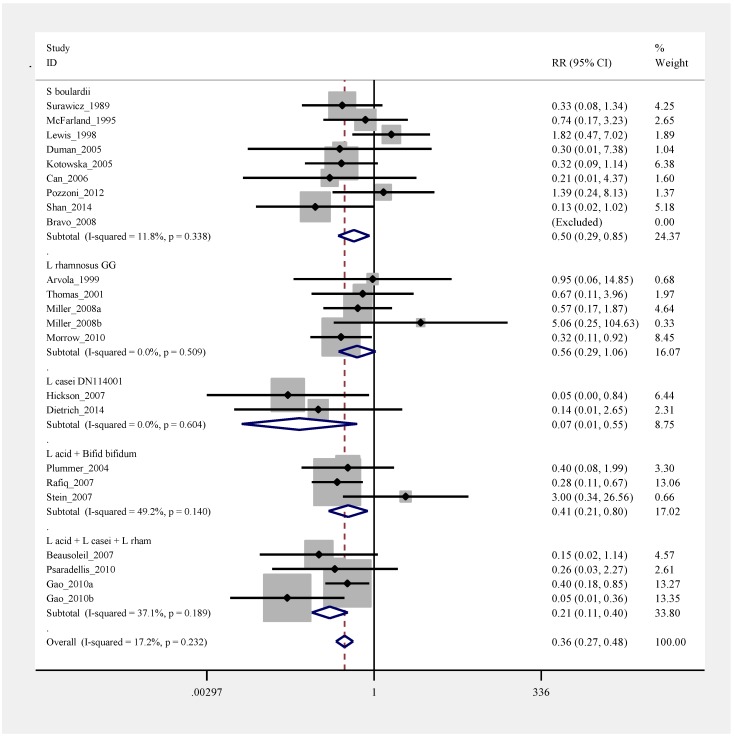
Forest plot of fixed effects model of meta-analysis of primary prevention of *C. difficile* disease by sub-group of probiotic type, x-axis indicates relative risk.

***Meta-analysis***. A meta-analysis of the 23 treatment arms of probiotic *versus* controls was performed and the pooled results indicated a low degree of heterogeneity (I^2^ = 17.2%, *p* = 0.23), so a fixed-effect model was used. As shown by the forest plot in Figure 2, when trials were pooled by similar types of probiotic species, four of five types of tested probiotic types were significantly effective for primary CDI prevention: *S. boulardii* (pRR = 0.50, 95% C.I. 0.29, 0.85), *L. casei* DN114001 (pRR = 0.07, 95% C.I. 0.01, 0.55), the mixture of *L. acidophilus* and *Bifido. bifidum* (pRR = 0.41, 95% C.I. 0.21, 0.80), and the mixture of *L. acidophilus* and *L. casei* and *L*. *rhamnosus* (pRR = 0.21, 95% C.I. 0.11, 0.40). The pooled results for *L. rhamnosus* GG did not reach statistical significance. A funnel plot (data not shown) and Egger’s text for publication bias did not show significant publication bias (*p* = 0.17).

***Sub-group analysis****.* Results from the meta-regression analysis for the adjunctive use of probiotics primary prevention of CDI did not find significant differences in associations between the study population (adult *versus* pediatric, *p* = 0.68), or daily dose of probiotic (≥10^10^ cfu/day *versus* <10^10^ cfu/day, *p* = 0.18). Only the probiotic strain group showed significance, confirming the validity of analyzing efficacy by strain type.

### 2.9. Pooled Efficacy of Probiotics for Secondary CDI Prevention

**Figure 3:**
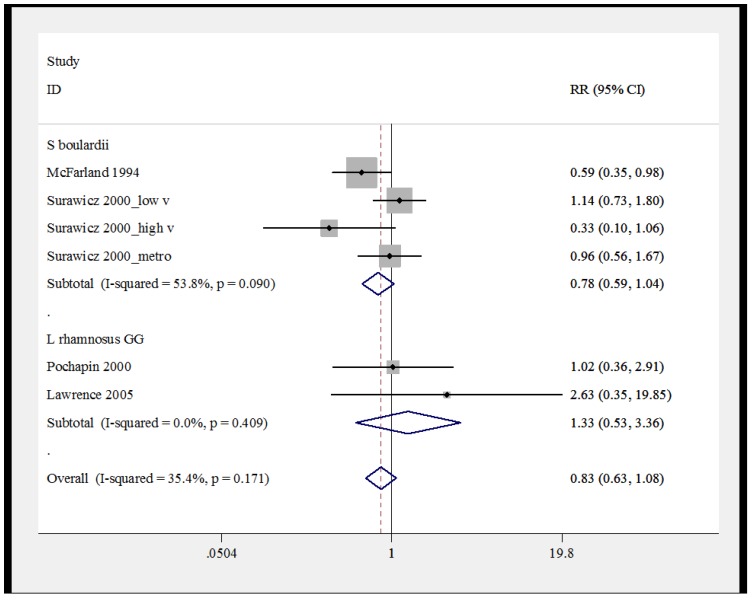
Forest plot of fixed effects model of meta-analysis of secondary prevention of *C. difficile* disease by sub-group of probiotic type, x-axis indicates relative risk.

***Meta-analysis***. A meta-analysis of the six treatment arms of probiotic *versus* controls was performed and the pooled results indicated a moderate degree of heterogeneity (I^2^ = 35.4%, *p* = 0.17), so a fixed-effect model was used. As shown by the forest plot in Figure 3, when trials were pooled by similar types of probiotic species, neither *S. boulardii* nor *L. rhamnosus* GG was significantly efficious for secondary CDI prevention. Publication bias was not assessed due to the limited number of available trials.

## 3. Discussion

Clinical recommendations for the use of probiotics in CDI disease has been limited by the scarcity of well-done, randomized controlled trials using CDI as their powered, primary outcome. Most of the evidence results from prevention of AAD trials, which include CDI only as a secondary outcome and did not consider this outcome when calculating the needed study size for their trials (52% had ≤10% power). As a consequence, most individual trials have not found statistically significant efficacy for probiotics and the prevention of CDI. This meta-analysis pooled the results of these trials, resulting in a significant increase in power and detected some (but not all) probiotic types were effective in preventing primary cases of CDI. The evidence for probiotics and the secondary prevention of CDI recurrences remains hampered by a lack of randomized, controlled trials.

As research on probiotics has evolved, the efficacy and mechanisms-of-action has been found to be highly strain-specific, requiring that dissimilar types of probiotics to be analyzed as separate sub-groups [44]. Previous meta-analyses on probiotics for the prevention of CDI done before these guidelines were established pooled dissimilar types of probiotic species in their analysis [5,45]. A recent meta-analysis chose to pool their main outcomes across probiotic species, based on the hypothesis that the efficacy should be similar, as the mechanisms-of-action is similar for different probiotics [46]. I would disagree with this hypothesis, as different probiotic strains can have different mechanisms-of-action and resulting degrees of efficacies [47]. Another recent meta-analysis did not separate the different types of probiotics in their nine included trials [48]. More recent meta-analyses have presented their results by probiotic sub-groups, but were not as comprehensive as this meta-analysis: One meta-analysis included 11 trials [6] and another was only done in five pediatric trials [49]. Another meta-analysis included 20 trials and did present pooled results by sub-groups, but the data was not presented within specific pooled probiotic groups [50].

The strengths of this meta-analysis include the extensive literature search of both established literature databases, use of grey literature and correspondence with experts in the field, inclusion of a large number of high to moderate quality randomized, controlled clinical trials, the analysis of the efficacy for both primary CDI prevention and secondary CDI prevention by probiotic type sub-groups and the use of standardized methods adhering to current meta-analytic guidelines. The result is a comprehensive evaluation of the types of probiotics that are effective in preventing CDI, allowing clinicians to evaluate whether the use of probiotics may be effective in their patients. Limitations of this meta-analysis are inherent in the reporting of published trials with missing data (for example, not all reported the types of antibiotics or the number of participants tested for *C. difficile*) and the limited number of confirmatory trials tested for each type of probiotic. Of the 15 different types of probiotics with randomized trials for the prevention of CDI, only five (33%) had multiple trials, allowing pooling of their results. More well-done trials need to be done testing the same types of probiotics.

## 4. Methods

### 4.1. Aims

The two aims of this review were to assess the use of specific probiotics for: (1) primary prevention of *C. difficile* disease (CDI) and (2) secondary prevention of *C. difficile* recurrences. Primary prevention of CDI is defined as people without diarrhea symptoms who are exposed to antibiotics and are given the intervention who do not develop diarrhea associated with a positive *C. difficile* assay (culture, immune assay, cytotoxin test or other assay) within two months exposure to the inciting antibiotic. Secondary prevention of CDI (prevention of CDI recurrences) is defined as people who have recovered from at least one prior episode of CDI, are asymptomatic (no diarrhea) at the time of the intervention and do not develop a recurrence of CDI within 1–2 months of follow-up.

### 4.2. Search Strategy

This meta-analysis followed PRISMA (Preferred Reporting Items for Systematic reviews and Meta-Analysis) statement guidelines [51] and guidelines using clearly delineated parameters, *a priori* inclusion and exclusion criteria and standardized data extraction tools [52,53] Systematic searches of PubMed (1960–2015), EMBASE (1974–2015), Cochrane Database of Systematic Reviews (1990–2015), ISI Web of Science (2000–2015) and three on-line clinical trial registries: Cochrane Central Register of Controlled trials (http://www.cochrane.org), MetaRegister of Controlled Trials (http:www.controlled-trials.com/mrct) and National Institutes of Health (http://www.clinicaltrials.gov) were done. All bibliographies from relevant studies were used to do a recursive search. Additional sources included: extensive grey literature search including abstracts from annual infectious disease and gastroenterology meetings, probiotic product websites, communication with experts in the field and published authors. Search terms included: *C. difficile* prevention, antibiotic-associated diarrhea, randomized controlled trials and specific probiotic strains. Search strategies were broad-based initially, then narrowed to the disease and population of interest. Abstracts of all citations and retrieved studies were reviewed and rated for inclusion. Full articles were retrieved if probiotics were given prevent diarrhea or treat *C. difficile* infections.

### 4.3. Inclusion and Exclusion Criteria

Inclusion criteria included randomized (well described or partially) controlled trials (RCT), blinded or open trials, in pediatric or adult populations (inpatient or outpatients), published in peer-reviewed journals or on clinical trial websites, or as meeting abstracts. Non-English language trials were translated and included whenever possible. Exclusion criteria included pre-clinical studies, safety, kinetic or formulation phase 2 studies, case reports or case series, duplicate reports, trials of unspecified types of probiotics, non-randomized trials, incomplete or no outcomes reported, or if translation could not be obtained. Probiotic strains with only one randomized controlled trial (lacking at least one other confirmatory trial) were also excluded.

### 4.4. Data Extraction

The data was extracted from a database from a previous meta-analysis on primary prevention and updated with recent publications, while secondary prevention articles were added [6]. For articles published in abstract form only or for any missing significant data in full articles, further information was sought by contacting authors or by the company manufacturing the probiotic product. Using a standardized data extraction form, the following data was systematically collected: authors, year of publication and journal, population data (age range, setting, types of antibiotic exposures, types of inciting diseases), study aims and outcomes, study methods (study design, eligibility criteria, sample size calculations, interim analysis, statistical methods used, recruitment methods, subgroup analysis done), randomization (method of randomization allocation, randomization method), degree of blinding (open, single or double), intervention data (probiotic strains used, daily dose, duration of treatment, duration of follow-up, type of control used, treatment concealment), types of *C. difficile* assays done, results (balanced randomization achieved, attrition rate and reasons, comparison of treatment groups by demographics, *etc.*, CONSORT flow-chart provided), outcome data [by group, intent-to-treat (ITT) or as-per-protocol (APP) analysis], safety data (adverse events reported by group), discussion points (limitations, generalizability and comparison of study results to published papers), clinical trial registration, location of protocol, and source of funding.

### 4.5. Interventions

Included trials had participants who were randomized to either a probiotic group or a control group. The type of control group may have included either a placebo (blinded study) or no treatment (open study). The type of probiotic intervention included probiotics in any formulation (e.g., capsule, sachet, tablets, drink, *etc.*). Trials investigating non-specific probiotics or yogurts (e.g., articles not providing the probiotic strain(s) used) were excluded. The most recent probiotic strain designations are presented in this study for those strains whose names have changed over time (older articles may have reported a different strain designation). The taxonomy of the probiotic strain type was confirmed by correspondence with authors or the manufacturing companies.

### 4.6. Statistical Analysis

Statistical analysis was performed using Stata software version 12 (Stata Corporation, College Station, Texas) to calculate pooled relative risks (pRR), bias estimates and number-needed-to-treat statistics. Univariate analysis results were analyzed using *X*^2^ test or Fisher’s exact test for small cell sizes (<5) with a significance level of *p* < 0.05. Meta-analysis was conducted for primary outcomes (CDI) using models to calculate the pooled relative risk and corresponding 95% confidence interval (95% CI) using the DerSimonian Laird method. Heterogeneity across trials was evaluated using Cochran Q test based on pooled relative risks by the Mantel-Haenazel method [54]. If the studies were homogenous, a fixed effects model was used; if studies were heterogeneous, a random effect model was employed. A *p*-value < 0.05 is considered statistically significant. The models used in this analysis were weighted by sample size, as study quality did not improve the fit.

If significant heterogeneity was found, subgroup analyses were conducted to determine the potential sources of heterogeneity. To explore possible explanations for heterogeneity, *a priori* subgroup analyses were conducted on study population (adult *versus* pediatric) and daily dose (≥ 1 × 10^10^ colony-forming units (cfu) per day or <1 × 10^10^ cfu/day). A meta-regression was done without the subgroup indicator and compared to a model with the subgroup indicator included. The difference in tau^2^ estimates from the two models indicates the proportion of study heterogeneity explained by the subgroup covariate (between study variance).

### 4.7. Publication Bias

To assess for publication bias, a funnel plot, as well as a weighted regression (Egger’s test) and a rank correlation test (Begg’s test for small study effects) were conducted [52,55]. Funnel plots show graphically that as sample sizes of trials increase, the precision is estimating the underlying treatment effect increases, which results in the effect estimates (relative risks) from small trials scattering more widely at the bottom of the graph and narrower scattering among larger studies. In the absence of publication bias, the funnel plot resembles a symmetrical inverted funnel. Reporting bias (smaller studies showing no protective effect) often are not published, and are indicated by an asymmetrical appearance with a gap in the bottom left of a funnel plot [56].

## 5. Conclusions

Four different types of probiotics were found to be effective for primary prevention of CDI (*S. boulardii*, *L. casei* DN114001, the mixture of *L. acidophilus* and *Bifido. bifidum* and the mixture of *L. acidophilus*, *L. casei* and *L. rhamnosus*). *L. rhamnosus* GG was not significantly efficious for the primary prevention of CDI and the other 10 types of probiotics lacked a second trial, so pooling of their outcomes was not possible. More clinical experience with these four probiotics might be recommended to confirm if they are effective in larger populations of patients.

Only two types of probiotics (*S. boulardii* and *L. rhamnosus* GG) had sufficient numbers of trials for to assess secondary prevention of CDI by meta-analysis, but none of the pooled results reached statistical significance. It may be that neither of these strains were effective in this analysis for preventing CDI recurrences, but based on prior experience and use of these probiotics (mechanism of action studies, case series, *etc.*), there are indications that these probiotic strains may be effective if an effective combination of probiotic and anti-*C. difficile* antibiotics can be determined [57,58].
